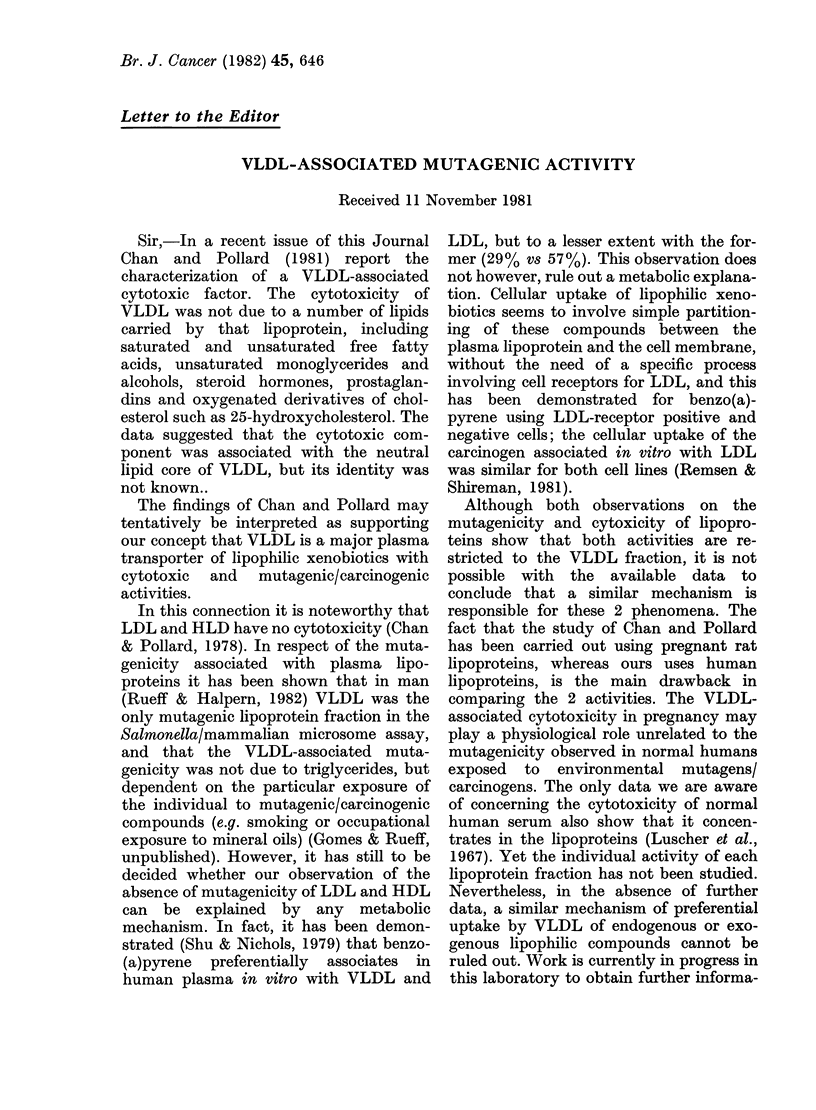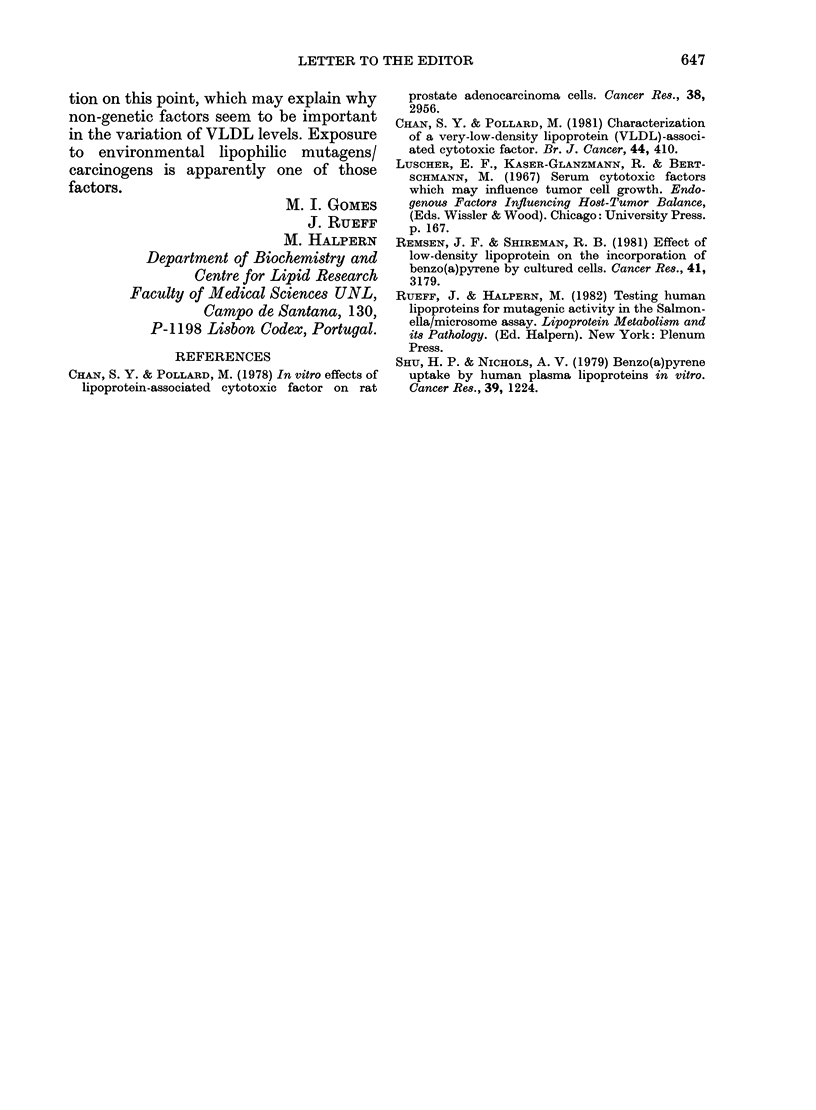# VLDL-associated mutagenic activity.

**DOI:** 10.1038/bjc.1982.103

**Published:** 1982-04

**Authors:** M. I. Gomes, J. Rueff, M. Halpern


*Br. J. Cancer* (1982) **45**, 646

*Letter to the Editor*

### VLDL-ASSOCIATED MUTAGENIC ACTIVITY

Received 11 November 1981

Sir,—In a recent issue of this Journal Chan and Pollard (1981) report the characterization of a VLDL-associated cytotoxic factor. The cytotoxicity of VLDL was not due to a number of lipids carried by that lipoprotein, including saturated and unsaturated free fatty acids, unsaturated monoglycerides and alcohols, steroid hormones, prostaglandins and oxygenated derivatives of cholesterol such as 25-hydroxycholesterol. The data suggested that the cytotoxic component was associated with the neutral lipid core of VLDL, but its identity was not known..

The findings of Chan and Pollard may tentatively be interpreted as supporting our concept that VLDL is a major plasma transporter of lipophilic xenobiotics with cytotoxic and mutagenic/carcinogenic activities.

In this connection it is noteworthy that LDL and HLD have no cytotoxicity (Chan & Pollard, 1978). In respect of the mutagenicity associated with plasma lipoproteins it has been shown that in man (Rueff & Halpern, 1982) VLDL was the only mutagenic lipoprotein fraction in the *Salmonella*/mammalian microsome assay, and that the VLDL-associated mutagenicity was not due to triglycerides, but dependent on the particular exposure of the individual to mutagenic/carcinogenic compounds (*e.g.* smoking or occupational exposure to mineral oils) (Gomes & Rueff, unpublished). However, it has still to be decided whether our observation of the absence of mutagenicity of LDL and HDL can be explained by any metabolic mechanism. In fact, it has been demonstrated (Shu & Nichols, 1979) that benzo(a)pyrene preferentially associates in human plasma *in vitro* with VLDL and LDL, but to a lesser extent with the former (29% *vs* 57%). This observation does not however, rule out a metabolic explanation. Cellular uptake of lipophilic xenobiotics seems to involve simple partitioning of these compounds between the plasma lipoprotein and the cell membrane, without the need of a specific process involving cell receptors for LDL, and this has been demonstrated for benzo(a)pyrene using LDL-receptor positive and negative cells; the cellular uptake of the carcinogen associated *in vitro* with LDL was similar for both cell lines (Remsen & Shireman, 1981).

Although both observations on the mutagenicity and cytoxicity of lipoproteins show that both activities are restricted to the VLDL fraction, it is not possible with the available data to conclude that a similar mechanism is responsible for these 2 phenomena. The fact that the study of Chan and Pollard has been carried out using pregnant rat lipoproteins, whereas ours uses human lipoproteins, is the main drawback in comparing the 2 activities. The VLDL-associated cytotoxicity in pregnancy may play a physiological role unrelated to the mutagenicity observed in normal humans exposed to environmental mutagens/ carcinogens. The only data we are aware of concerning the cytotoxicity of normal human serum also show that it concentrates in the lipoproteins (Luscher *et al.*, 1967). Yet the individual activity of each lipoprotein fraction has not been studied. Nevertheless, in the absence of further data, a similar mechanism of preferential uptake by VLDL of endogenous or exogenous lipophilic compounds cannot be ruled out. Work is currently in progress in this laboratory to obtain further information on this point, which may explain why non-genetic factors seem to be important in the variation of VLDL levels. Exposure to environmental lipophilic mutagens/carcinogens is apparently one of those factors.

M. I. GOMES
J. RUEFF
M. HALPERN
*Department of Biochemistry and Centre for Lipid Research*
*Faculty of Medical Sciences UNL,*
*Campo de Santana, 130,*
*P-1198 Lisbon Codex, Portugal.*

REFERENCES

CHAN, S. Y. & POLLARD, M. (1978) *In vitro* effects of lipoprotein-associated cytotoxic factor on rat prostate adenocarcinoma cells. *Cancer Res.*, **38**, 2956.

CHAN, S. Y. & POLLARD, M. (1981) Characterization of a very-low-density lipoprotein (VLDL)-associated cytotoxic factor. *Br. J. Cancer*, **44**, 410.

LUSCHER, E. F., KASER-GLANZMANN, R. & BERTSCHMANN, M. (1967) Serum cytotoxic factors which may influence tumor cell growth. *Endogenous Factors Influencing Host-Tumor Balance*, (Eds. Wissler & Wood). Chicago: University Press. p. 167.

REMSEN, J. F. & SHIREMAN, R. B. (1981) Effect of low-density lipoprotein on the incorporation of benzo(a)pyrene by cultured cells. *Cancer Res.*, **41**, 3179.

RUEFF, J. & HALPERN, M. (1982) Testing human lipoproteins for mutagenic activity in the Salmonella/microsome assay. *Lipoprotein Metabolism and its Pathology.* (Ed. Halpern). New York: Plenum Press.

SHU, H. P. & NICHOLS, A. V. (1979) Benzo(a)pyrene uptake by human plasma lipoproteins *in vitro*. *Cancer Res.*, **39**, 1224.